# Role of the gut microbiome in psychological symptoms associated with inflammatory bowel diseases

**DOI:** 10.1007/s00281-025-01036-x

**Published:** 2025-01-27

**Authors:** Konstantina Atanasova, Laura-Louise Knödler, Wolfgang Reindl, Matthias Philip Ebert, Anne Kerstin Thomann

**Affiliations:** 1 Department of Medicine II, Medical Faculty Mannheim, University Medical Center Mannheim, Heidelberg University, Mannheim, Germany; 2 Department of Psychosomatic Medicine, Medical Faculty Mannheim, Central Institute for Mental Health Mannheim, Heidelberg University, Mannheim, Germany

**Keywords:** Gut microbiome, Brain-gut axis, Inflammatory bowel disease, Depression, Fatigue, Anxiety

## Abstract

The brain-gut axis constitutes the basis for the bidirectional communication between the central nervous system and the gastrointestinal tract driven by neural, hormonal, metabolic, immunological, and microbial signals. Alterations in the gut microbiome composition as observed in inflammatory bowel diseases can modulate brain function and emerging empirical evidence has indicated that interactions among the brain-gut microbiome-axis seem to play a significant role in the pathogenesis of both inflammatory bowel diseases and psychiatric disorders and their comorbidity. Yet, the immunological and molecular mechanisms underlying the co-occurrence of inflammatory bowel diseases and psychological symptoms are still poorly understood. The aim of this narrative review is to highlight contemporary empirical findings supporting a pivotal role of the gut microbiome in the pathophysiology of highly prevalent neuropsychiatric symptoms in inflammatory bowel diseases such as fatigue, depression, and anxiety. Finally, we focus on microbiome modulation as potential treatment option for comorbid neuropsychiatric symptoms in immune-mediated diseases and especially in inflammatory bowel diseases. High-quality clinical trials are required to clarify how microbiome modulation through dietary interventions or probiotic, prebiotic or synbiotic treatment can be used clinically to improve mental health and thus quality of life of patients with inflammatory bowel diseases.

**Supplementary Information:**

The online version contains supplementary material available at 10.1007/s00281-025-01036-x.

## Introduction

Inflammatory bowel diseases (IBD), comprising Crohn’s disease (CD) and ulcerative colitis (UC), are chronic immune-mediated conditions, characterized by recurrent inflammation of the gastrointestinal tract. Typical symptoms include diarrhea, abdominal pain and rectal bleeding. However, fatigue or symptoms of depression and anxiety constitute common extraintestinal complaints in IBD that do not directly relate to the gastrointestinal tract.

With around 50 to 80% of the patients experiencing symptoms of fatigue and depression during active disease, psychiatric symptoms constitute one of the most burdensome symptoms in IBD [1, 2]. The link between IBD and psychiatric disorders (meta-analyses: [3, 4]; systematic review: [2, 5]) as well as between IBD and the gut microbiome [6–8] has been extensively investigated in the past. Moreover, increasing evidence links the gut microbiome to a number of psychiatric disorders and fatigue [9, 10]. These relationships suggest that disturbances along the brain-gut-microbiome axis may at least partly explain the high comorbidity between gastrointestinal diseases and neuropsychiatric symptoms or disorders [11–13]. Despite the high prevalence of psychiatric comorbidities in IBD patients and their detrimental effect on patients’ quality of life, studies investigating alterations in the gut microbiome or/and the brain-gut-microbiome-axis as underlying mechanisms for the pathophysiology of mental health disorders in IBD are sparse. In this narrative review, we conducted a comprehensive literature research on the role of the gut microbiome as mediator for psychological symptoms such as fatigue, depression and anxiety in IBD patients and discuss the potential of gut microbiome modulation (e.g., via probiotics) for the management of mental health issues in IBD.

## Methods

### Search strategy

We conducted a literature research in Pubmed and OVID Embase (1910 to Present); MEDLINE(R) (1946 to Present with Daily Update, Epub Ahead of Print, In-Process & Other Non-Indexed Citations). The following keywords were used for two separate literature researches: (1) “Mental health”, “Fatigue”, “Depressive disorder”, “Anxiety disorders”, “Gastrointestinal microbiome”, “Brain-gut-axis”, “Inflammatory Bowel Diseases”, “Crohn Disease”, “Ulcerative Colitis”; (2) “Mental health”, “Fatigue”, “Depressive disorder”, “Anxiety disorders”, “Gastrointestinal microbiome”, “Brain-gut-axis”, “Inflammatory Bowel Diseases”, “Prebiotics”, “Probiotics”, “Synbiotics”, “Therapeutics”. Titles and abstracts were required to be published in a peer-reviewed journal and written in English. Articles published before 2015 as well as systematic reviews and meta-analyses were excluded. Further, conference abstracts, commentaries and articles with no full text available were excluded.

## Results

### Study selection

**Fig. 1:**
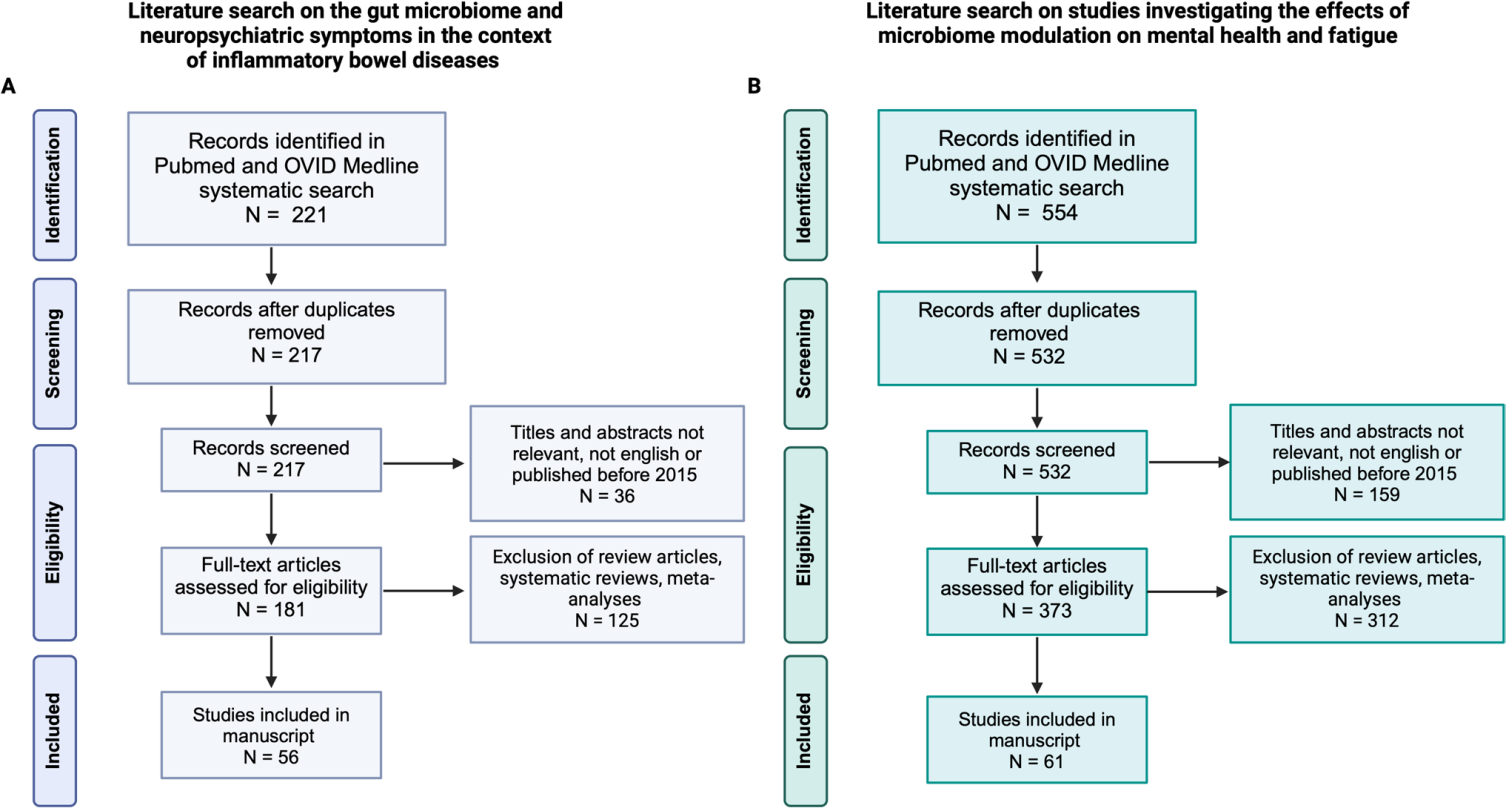
Prisma Flow Diagram visualizing the selection and filtering process of the conducted systematic literature search. (**A**) Studies investigating the role of gut microbiome in the link between inflammatory bowel disease and mental health; (**B**) Studies investigating pro-, pre- and synbiotic treatment in mental health disorders and inflammatory bowel disease

Overall, 221 and 554 article abstracts were retrieved in search (1) and (2), respectively. The selection and filtering processes are depicted in Fig. 1. Table 1 provides a summary of available randomized controlled trials on the effect of probiotic, prebiotic and synbiotic supplementation on mental health in humans. Studies investigating these effects in animals are summarized in Table S1, suppl. material.

**Table 1:** Overview of randomized clinical trials investigating the impact of probiotic, prebiotic and synbiotic treatment on psychological symptoms in healthy and clinical populations. ASD = Autism spectrum disorder, CFS = chronic fatigue syndrome, CT = clinical trial, IBS = irritable bowel syndrome, MDD = major depressive disorder

Reference	Intervention type	Study population	n	Probiotic/Prebiotic	Treatment duration	Main findings
Akkasheh et al. [14]	Randomized, double-blind, placebo-controlled CT	Patients with MDD	40	*Lactobacillus acidophilus, Lacticaseibacillus casei,* and* Bifidobacterium bifidum*	8 weeks	Reduction of depressive symptoms
Allen et al. [15]	Placebo-controlled CT	Healthy volunteers	22	*Bifidobacterium longum*	8 weeks + 2 weeks post-probiotic follow-up	Reduction of daily stress perception
Benton et al. [16]	Randomized, double-blind, placebo-controlled CT	Healthy volunteers	132	*Lacticaseibacillus casei* *Shirota*	3 weeks	Improved mood in those individuals whose mood was initially poor at baseline
Chong et al. [17]	Randomized, double-blind, placebo-controlled CT	Patients with stress	111	*Lactobacillus plantarum*	12 weeks	Reduced stress and anxiety levels
Freijy et al. [18]	Randomized, placebo-controlled, superiority trial	Adults with psychological distress	119	*Bifidobacterium bifidum, Bifidobacterium animalis subsp. Lactis, Bifidobacterium longum, Lactobacillus acidophilus, Lactobacillus helveticus, Lacticaseibacillus casei, Lactobacillus plantarum, Lactobacillus rhamnosus* *High-prebiotic diet*	8 weeks	No positive effects on mood, anxiety, depression, stress or wellbeing Improved anxiety, stress and sleep
Hadi et al. [19]	Randomized, double-blind, placebo-controlled CT	Adults with overweight or obesity	59	*Lactobacillus acidophilus, Lacticaseibacillus casei, Bifidobacterium bifidum* + inulin	8 weeks	Decreased stress perception, anxiety, depression
Haghighat et al. [20]	Randomized, double-blind, placebo-controlled CT	Hemodialysis patients	75	*Lactobacillus acidophilus, Bifidobacterium bifidum, Bifidobacterium lactis, Bifidobacterium longum*	12 weeks	Decrease in depression, decrease in anxiety
Kazemi et al. [21]	Randomized, double-blind, placebo-controlled CT	Patients with MDD	81	*Lactobacillus helveticus* and *Bifidobacterium longum*; *Galactooligosaccharide*	8 weeks	Reduction of depressive symptoms
Kelly et al. [22]	Randomized, placebo-controlled, cross-over CT	Healthy male volunteers	29	*Lactobacillus rhamnosus*	8 weeks	No effects on mood, anxiety, stress or sleep quality
Lau et al. [23]	Randomized, double-blind, placebo-controlled CT	Patients with post-acute Covid-19 syndrome	463	*Bifidobacterium adolescentis, Bifidobacterium bifidum, Bifidobacterium longum* + alacto- oligosaccharides, xylo-oligosaccharides, and resistant dextrin	6 months	Reduction of fatigue symptoms, improved general wellness and cognitive abilities (memory, concentration)
Lorenzo-Zúñiga et al. [24]	Multicenter, randomized, double-blind, placebo-controlled CT	IBS patients	84	*Lactobacillus plantarum* and *Pediococcus acidilactici*	6 weeks	Reduction of gastrointestinal specific anxiety
Majeed et al. [25]	Randomized, double-blind, placebo-controlled CT	IBS patients with MDD	40	*Bacillus coagulans*	90 days	Reduction of depressive and IBS-related symptoms
Messaoudi et al. [26]	Randomized, double-blind, placebo-controlled CT	Healthy volunteers	55	*Lactobacillus helveticus* and *Bifidobacterium longum*	4 weeks	Decreased levels of somatisation, depression and anxiety
Messaoudi et al. [27]	Secondary analysis of a subsample from [26]	Healthy volunteers	25	*Lactobacillus helveticus* and *Bifidobacterium longum*	4 weeks	Reduction of symptoms of depression, anxiety and perceived stress
Mysonhimer et al. [28]	Randomized, placebo-controlled, single-blind, crossover CT	Healthy volunteers	24	Lactaidlow-fat 1% milk, fructooligosaccharide, galactooligosaccharide	4 weeks	No differences between prebiotic treatment and placebo regarding depression, anxiety, emotion perception or sleep quality
Obermoser et al. [29]	Randomized, double-blind, placebo-controlled pilot study	Patients with post-acute Covid-19 syndrome	70	*Lacticaseibacillus casei, Lactobacillus acidophilus, Lactobacillus paracasei, Bifidobacterium lactis, Lactobacillus salivarius, Lactococcus lactis, Bifidobacterium lactis, Lactobacillus plantarum, Bifidobacterium bifidum*	6 months	Reduction of fatigue and depression symptoms
Östlund-Lagerström et al. [30]	Randomized, double-blind, placebo-controlled CT	Older adults (> 65 years)	290	*Lactobacillus reuteri*	12 weeks + 8 & 12 weeks follow-up	Reduction of perceived stress; reduction of anxiety in individuals with gastrointestinal complaints
Pinto-Sanchez et al. [31]	Randomized, double-blind, placebo-controlled CT	IBS patients	44	*Bifidobacterium longum*	6 weeks + 4 weeks follow-up	Reduction of depression symptoms and improvement of quality of life
Rao et al. [32]	Randomized, double-blind, placebo-controlled pilot study	CFS	39	*Lacticaseibacillus casei*	8 weeks	Reduction of anxiety symptoms
Romijn et al. [33]	Randomized, double-blind, placebo-controlled CT	Persons with depressive symptoms (no MDD)	69	*Lactobacillus helveticus, Bifidobacterium longum*	8 weeks	No significant effects of PRO on mood
Schmidt et al. [34]	Randomized, double-blind, placebo-controlled CT	Healthy volunteers	45	*Galacto-oligosaccharide/fructo-oligosaccharide*	3 weeks	Reduction of waking cortisol levels and psychological distress
Steenbergen et al. [35]	Randomized, triple-blind, placebo-controlled, pre- and post-intervention CT	Healthy volunteers	40	*Bifidobacterium bifidum, Bifidobacterium lactis, Lactobacillus acidophilus, Lactobacillus brevis, Lacticaseibacillus casei, Lactobacillus salivarius, Lactococcus lactis*	4 weeks	Reduced rumination and negative thoughts
Vidot et al. [36]	Randomized, placebo-controlled pilot study	Patients with hepatic encephalopathy	49	Lactobacillus paracasei ssp paracasei*, Lactobacillus plantarum, Leuconostoc mesenteroides, Pediococcus pentosaceus* + fibres + branched chain amino acids (Hepatamine®)	8 weeks	Decreased anxiety scores, no effects on depression and stress perception

## Discussion

### Linking neuropsychiatric symptoms in IBD to the microbiome

#### Depression and IBD: role of the gut microbiome

Chronic medical conditions and autoimmune disorders constitute a risk factor for depression and anxiety, indicating that inflammatory processes play a crucial role in the pathophysiology of mood disorders [37, 38]. Previous studies have demonstrated a link between depression and upregulated immune activity in IBD, which supports the hypothesis of a common immune-mediated pathway for depressive disorders and somatic diseases with inflammatory origin [39–42].

From a clinical point of view, episodes of major depression are linked to dysregulated functioning of the hypothalamic–pituitary–adrenal (HPA) axis, which regulates the adaptive stress response in the body [43]. Dysregulations of the HPA axis signaling have been linked to the pathophysiology of mood disorders, being typically associated with higher levels of cortisol and inflammatory mediators that result in a sustained pro-inflammatory state [44]. The presence of a commensal microbiome is crucial to numerous aspects of host physiology including immune functioning, nutrient processing and functioning of the central nervous system (CNS) [45–48]. Animal research has demonstrated a direct link between HPA reactivity and the gut microbiome, showing an exaggerated corticosterone and adrenocorticotrophin response to stress in germ-free (GF) mice compared to conventionally pathogen-free mice [49]. GF show no commensal microbiome composition and an undeveloped immune system, hallmarked by increased stress reactivity to environmental stressors [46, 50]. Stress is known to increase intestinal permeability, making it possible for bacteria to translocate across the intestinal mucosa and directly access both immune and neuronal cells of the enteric nervous system [51, 52].

Several studies explored the mechanisms underlying the association between the gut microbiome and the HPA axis [43, 53, 54]. One hypothesis suggests that gut dysbiosis may contribute to enhanced cytokines release (e.g., IL-1β, IL-6, TNF-α), resulting in a higher activation of the HPA axis and enhancing the risk of developing symptoms of anxiety and depression in IBD [53, 55–57]. Although there is strong evidence from rodent studies that gut microbiome has an essential impact on the HPA axis functioning and vice versa, human studies exploring the mediating role of the HPA axis in the link between psychiatric disorders and gut dysbiosis are still lacking.

The bi-directional communication between the gut microbiome and the brain is highly complex and comprises an immune pathway, tryptophan [58] metabolism, vagus nerve activity, and the enteric nervous system, as well as metabolites produced by the gut microbiome [59–62]. Short-chain fatty acids (SCFAs) are microbially derived metabolites, which are products of bacterial fermentation. More than 95% of SCFAs produced in the gut are acetate, propionate and butyrate [63]. With respect to the pathophysiology of depression, alterations in the gut microbiome composition have been widely reported in the literature (for systematic review: [64–67]), demonstrating higher abundance of pro-inflammatory species (e.g., *Enterobacteriaceae*) [68] and lower levels of SCFAs producing bacteria (e.g., *Faecalibacterium*) [69] being associated with more severe depressive symptoms.

Given the established connection between gut dysbiosis and IBD on one hand and the microbiome and psychiatric symptoms or disorders on the other, a mediating if not causal role of the gut microbiome in the development of psychiatric symptoms and disorders in IBD seems likely [70–72]. Qin et al. recently demonstrated several significant associations between an IBD diagnosis and specific changes in the gut microbiome, being linked to the presence of depressive symptoms [73]. Here, depression was associated with altered abundances of *Clostridium*, *Desulfovibrionaceae*, *Ruminococcaceae* and *Akkermansia* and changes within the class of Deltaproteobacteria and the family *Ruminococcaceae*, dependent on patients’ age. Further studies on this topic demonstrated a reduced alpha diversity to be associated with higher depression scores in CD [74, 75]. In CD, higher depression scores were linked to reduced relative abundances of *Eubacterium*, *Clostridiales, Lachnospiraceae, Roseburia*, and *Ruminococcus,* as well as to higher abundance of *Bifidobacterium* [74]. However, another pattern was observed for UC patients, with higher depression scores being associated with reduced occurrence of *Erysipelotrichaceae, Lachnospira, Blautia, Phascolarctobacterium*, and *Streptococcus* and higher abundance of *Desulfovibrio* [74]. In contrast, Scaldaferri et al. found an increase in *Blautia* and *Streptococcus* to be most strongly associated with depressive symptoms in IBD [76]. Several studies found that the genus *Streptococcus* is increased in patients with depressive disorder (for review: [66, 77]), and it has been found to produce serotonin [78], indicating its potential role in the pathophysiology of depression in IBD. However, since findings seem to be inconclusive on the exact role of *Streptococcus* in the link between IBD and depression, further research is needed. Depressive symptoms in patients with UC were further associated with a decreased abundance of *Enterobacteriaceae* [76]. The family *Enterobacteriaceae* is essential for the production of SCFAs through the fermentation of carbohydrates. This process induces the biosynthesis of serotonin on the level of enterochromaffin cells, which upon activation, release serotonin to act upon serotonin receptors on neurons in the enteric nervous system [79, 80]. Thus, a reduction of the *Enterobacteriaceae* family might result in lower levels of serotonin and therefore, in more severe depressive symptoms in IBD patients [81, 82]. However, as empirical evidence is still inconclusive and several studies have shown an increase of *Enterobacteriaceae* in patients with major depressive disorder [64, 83], further studies on the role of *Enterobacteriaceae* in the pathophysiology of depression are needed. Finally, Scaldaferri et al. also reported alterations in the genus *Veillonella*, being associated with both depressive and anxious symptoms in IBD, a pattern already observed in individuals with depression. *Veillonella* is one of the bacteria with lipopolysaccharides at the outer membrane level [84], which may stimulate the release of pro-inflammatory cytokines, such as IL-6 [85, 86], potentially promoting the development of depressive symptoms through the immune-mediated pathway.

#### Anxiety and IBD: role of the gut microbiome

With respect to anxiety symptoms, reduced alpha diversity and reduced abundances of *Fusobacterium* were found to be linked to more severe anxiety symptoms in UC patients [74, 75]. Humbel et al. reported several taxonomic changes in *Bacillota, Bacteroidota* and *Pseudomonadota*, with reduced abundances of species belonging to these phyla being associated with stronger anxiety symptoms in patients with IBD [74]. It has been shown that fecal microbiome transplantation can promote mental health in individuals with irritable bowel syndrome (IBS) by improving the relative abundance of the beneficial *Bacteroidota* and suppressing the pathogenic releaser *Enterobacteriaceae* [87]. In line, Scaldaferri et al. found an increase in *Enterobacteriaceae* to be associated with stronger anxiety symptoms in IBD [76], a link already demonstrated in patients with generalized anxiety disorder [88]. A recent study by Joo et al. [89] demonstrated a positive association between *Enterobacterales_f* and *Enterococcaceae* and anxiety and depression in patients with UC. Oral administration of *Enterococcus mundtii*, belonging to *Enterococcaceae*, resulted in reduced levels of anti-depressive neuropeptide Y in the colon, plasma, and hippocampus, leading to anxiety and depression-like behavior in mice. Therefore, it could be speculated that the pathogenicity of Enterococci may be associated with a direct or indirect effect of *Enterococcus mundtii* on both, the enteric and central nervous system, resulting in a higher risk of affective disorders such as anxiety and depression [89].

#### Fatigue in IBD: role of the gut microbiome

Symptoms of fatigue frequently occur in immune-mediated diseases and encompass a substantial reduction or impairment in the ability to engage in daily activities, cognitive impairments and sleep disturbances. Various studies have demonstrated a strong comorbidity of depression and fatigue, which can be partially explained by shared immune-inflammatory pathways, including increased translocation of Gram-negative bacteria and increased levels of pro-inflammatory cytokines (for review: [90, 91]). The high prevalence of fatigue symptoms in IBD is well acknowledged, but poorly understood [92]. One theory states that it is caused by a subclinical inflammatory state characterized by elevated levels of circulation cytokines in the absence of overt symptoms of inflammation [2]. Vogelaar et al. [93] could demonstrate that serum levels of IL-12 and IL-10 were increased, as were stimulated TNF and IFN-γ levels, while serum IL-6 levels were lower in IBD patients reporting severe fatigue symptoms. Moreover, fatigue was linked to lower IL-2 serum levels and granulocyte-monocyte colony stimulating factor in IBD [94]. Other studies found no differences in serum levels of IL-5, IL-8, and IL-12 between patients with and without fatigue [95] and indicated that markers of subclinical inflammation could not be used to discriminate between different severities of fatigue during remission [96]. Recent evidence indicates that in remitted IBD fatigue symptoms are not likely to be primarily driven by systemic inflammatory activity [97].

Another pathway that may contribute to the pathogenesis of fatigue in IBD is controlled by tryptophan metabolism, which has been shown to be altered in IBD. During active phases of the disease patients with IBD exhibit decreased tryptophan levels mainly as a result of an inflammation-induced upregulation of indoleamine 2,3-dioxygenase (IDO1), which is a rate-limiting enzyme catalyzing tryptophan to kynurenine and shifting tryptophan metabolism to the kynurenine pathway [98]. Low serum tryptophan levels have been associated with IBD-related fatigue, indicating the potential of tryptophan supplementation as treatment for fatigue in IBD [99]. While this was not effective to reduce fatigue in remitted IBD [100], it has not been investigated in active disease, where decreased levels of tryptophan may be more evident as a reason for symptoms of fatigue. Some previous studies have also explored the role of quinolinic acid, a downstream product of the kynurenine pathway, in the pathophysiology of psychiatric disorders [101–103]. Dysregulations of the tryptophan-to-serotonin pathway have been previously linked to more severe symptoms of fatigue in other disorders, and treatment with 5-hydroxytryptophan (5-HTP) showed positive effects on fatigue in patients with fibromyalgia [104].

Fatigue has a multifactorial pathophysiology, and alterations in the gut microbiome composition have been considered to play a crucial role in the development of fatigue in IBD. Studies demonstrated an altered microbiome composition in myalgic encephalomyelitis/chronic fatigue syndrome (CFS), characterized by a decreased abundance of *Actinomycetota* and depletion of *Bacillota* [105–107], a pattern observed also in active IBD [108, 109]. A recent multi-omic study has demonstrated a reduced abundance of *Faecalibacterium prausnitzii* and *Lactobacillus rogosae* being associated with more severe fatigue symptoms in patients with chronic fatigue syndrome [110]. *Faecalibacterium prausnitzii,* a butyrate-producing bacterium, can exert anti-inflammatory effects in the intestine through its production of microbial anti-inflammatory molecules and salicylic acid. Borren et al. have shown reduced levels of *Faecalibacterium prausnitzii* in IBD patients with fatigue compared with these without fatigue [99], supporting the role of this bacterium in fatigue symptoms in immune-mediated conditions, possibly via SCFA signalling. Another study on the link between the gut microbiome and fatigue in IBD found a lower alpha diversity and reduced abundance of *Ruminococcus*, *Faecalibacterium, Alistipes* and *Roseburia* bacteria to be associated with fatigue symptoms in remitted IBD [99]. As described above, bacteria belonging to the genera of *Faecalibaterium* and also *Roseburia* are also known to produce butyrate, a SCFA that is a product of dietary carbohydrates transformed by gut bacteria [62]. SCFAs contribute to maintaining the mucosal barrier and seem to downregulate pro-inflammatory cytokines, having immunomodulatory and anti-inflammatory effects for the organism, underlying their potential to mediate or moderate fatigue in IBD. The study by Borren et al. further describes alterations alterations in the serum metabolome underpinned by several alterations in the gut microbiome, including decreased tryptophan and a depletion of butyrate-producing phyla [99]. The authors interpreted these findings as a potential indicator for a local pro-inflammatory environment in the gut that is related to fatigue even if clinical and endoscopic markers indicate a remitted stage of the disease [99].

Previous work of our research group found moderate to strong associations between the four genera *Intestinimonas, Anaerotruncus, Eubacterium* and *Clostridiales g.i.s* and fatigue symptoms in IBD within a triangular association between symptoms, bacterial genus abundances and microbial metabolic pathways [75]. Higher fatigue scores were linked to a decreased abundance of *Intestinimonas*, bacteria that have the ability to degrade Amadori products (fructosamines) and especially fructoselysine into the SCFA butyrate, while fructosamines can be used by other bacteria, e.g. *E. coli* as a source for glucose. Although the link between these pathways and fatigue is speculative, both SCFA and amino acids have neuromodulatory features that may mediate between the gut microbiome and fatigue symptoms [75, 99, 110].

**Fig. 2:**
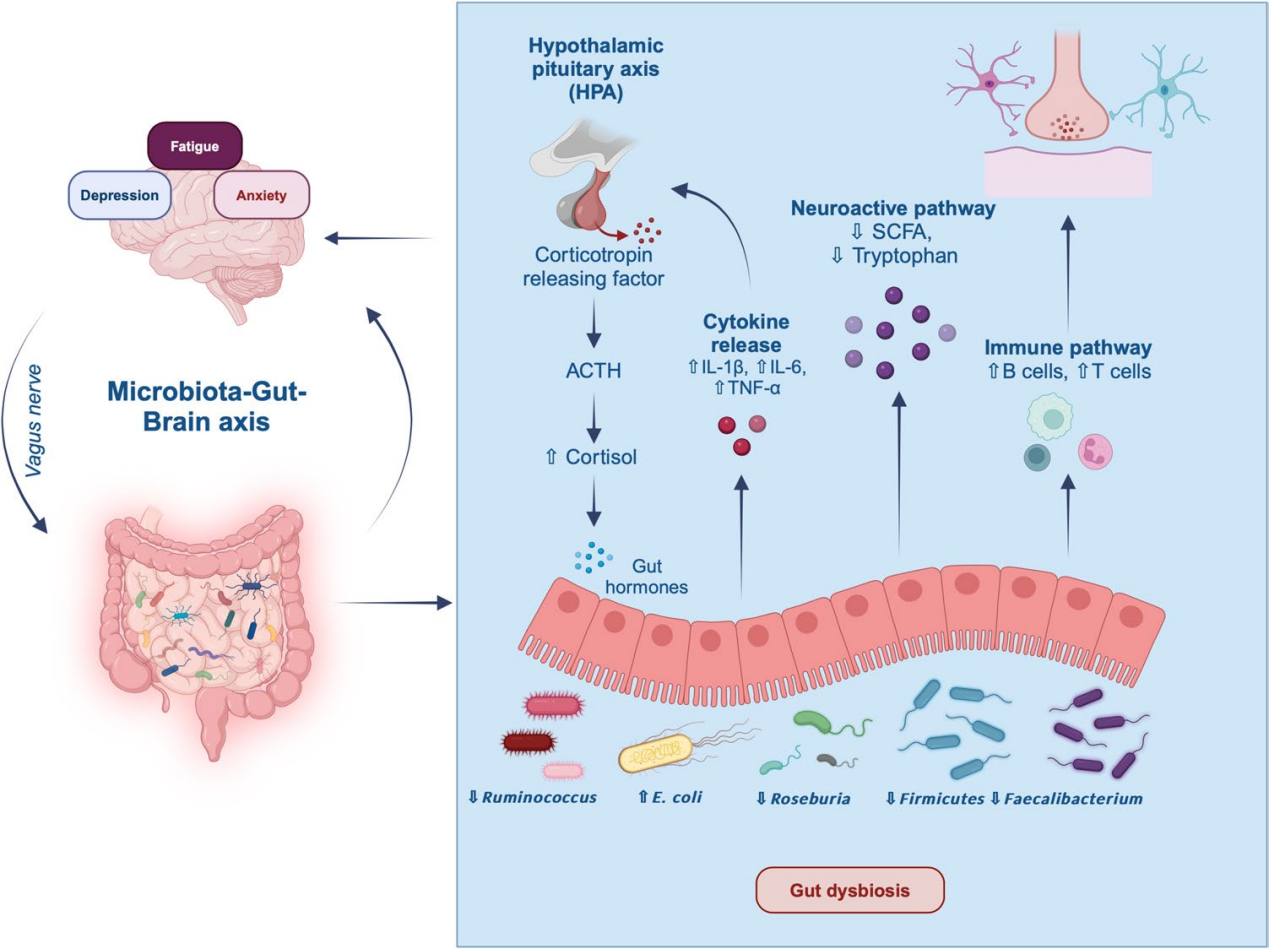
Communication pathways between the brain and gut microbiome in IBD. Comorbid psychological symptoms such as depression, anxiety and fatigue have been linked to altered gut microbiome and gut dysbiosis in IBD. Figure created in https://BioRender.com

Taken together, the described research emphasizes the special role of SCFA-producing gut bacteria in the pathophysiology of neuropsychiatric symptoms in IBD. These findings indicate the potential of interventions targeting a microbiome modulation such as pre- and probiotics to improve psychological symptoms such as fatigue in IBD. Figure 2 illustrates biological and psychological factors as well as mechanisms of interaction along the microbiota-gut-brain-axis in IBD, potentially underlying the development of symptoms of depression, anxiety and fatigue.

### Microbiome modulation and mental health in IBD: chances and challenges

#### Nutrition

Nutrition plays a crucial role in physical and mental health and certain foods belonging to a sugar- and fat-rich Western diet (e.g., refined carbohydrates) have been shown to have detrimental effects on the gut microbiome, promoting microbes associated with an increased risk of developing IBD [111–113]. The term immunonutrition refers to the effects that dietary factors can have on multiple aspects of the immune system as well as the gut microbiome [114, 115]. Within the gastrointestinal tract, nutrients are likely to affect mucosal barrier function and cellular defense and modulate local inflammatory processes [116–119]. A nutritionally balanced diet is essential for the maintenance of a healthy gut microbiome, the integrity of the intestinal barrier, and immune tolerance [120], while an unbalanced diet like the typical western diet, characterized by high amount of refined grains, processed foods and sugars, results in reduced microbial diversity, increasing the risk of a leaky gut and chronic inflammation [121–125]. In individuals consuming a healthy, balanced diet (rich in high-fiber vegetables, whole grains, omega-3 foods), intestinal homeostasis seems to be maintained through the release of several microbial metabolites, including SCFAs, improving the intestinal barrier function by providing energy for colonic epithelial cells and promoting regulatory T cell function [126, 127]. SCFAs are used by colonocytes or absorbed into the systemic circulation where they bind to the G protein-coupled receptors GPR41 and GPR43, mediating protective immunity by promoting epithelial cell production of cytokines and chemokines [128]. Butyrate as one of the best studied SCFAs acts as a key source of energy for colonocytes and possesses powerful anti-inflammatory features [129], exhibiting immunoregulatory, anti-obesity, anti-diabetes, cardiovascular protective and neuroprotective effects (for review: [130]). Further, regulation of SCFA levels was also introduced as a pathomechanistic link between the gut microbiome and major depression [68, 131–133], emphasizing the potential role of microbiome modulation in the treatment of affective disorders. Multi-omics analyses have demonstrated positive effects of specific bacteria on mental health, including *Bacteroides uniformis, Roseburia inulinivorans, Eubacterium rectale*, and *Faecalibacterium prausnitzii* [65, 134]*.* These have not only been associated with the production of SCFA, but also the regulation of amino acids, taurine, and cortisol metabolism, biological processes strongly related to the pathophysiology of depression, anxiety and fatigue [135–138].

An unhealthy diet can promote altered intestinal immune system responses through a gut microbiome dysbiosis, resulting in disturbances of the intestinal barrier function, system inflammation, altered glucose metabolism, and a state of low-grade chronic mucosal inflammation [124, 139, 140]. Recent studies have indicated the key role for epithelial cell metabolism in controlling the gut microbiome, especially the overgrowth of *E. coli* [141, 142]. Under healthy conditions, colonocytes use butyrate as major energy source. Metabolizing butyrate consumes oxygen and thus, rendering surface colonocytes hypoxic and promoting the luminal growth of strict anerobic bacteria such as *Bacillota* [143, 144]. On the other side, intestinal inflammation leads to a loss of *Bacillota*, resulting in a reduced butyrate production. In case of absence of butyrate, colonocytes obtain energy through the fermentation of glucose to lactate, a process increasing oxygen levels in these cells. The increased amount of oxygen in colonocytes significantly affects the local microbes, leading to a depletion of strict anaerobes while favoring the expansion of facultative anaerobic bacteria such as *E. coli* pathobionts as observed in the gut microbiome of patients with IBD [145, 146].

Taken together, the potential role of a balanced diet rich in fiber and fermented foods to improve somatic and mental health in IBD cannot be stressed enough, but needs to be further endorsed by high-quality clinical studies.

#### Pro- and prebiotics

Microbial dysbiosis in IBD could be overcome using delivery of beneficial gut microbes (probiotics) known to be lacking or of reduced abundance in this chronic condition, and probiotics have repeatedly shown a certain efficacy in the treatment of IBD-related inflammation [147–150]. Probiotics can be easily incorporated into patients’ diet through the consumption of fermented foods such as yoghurt, kefir, kimchi, or consumed as a probiotic supplement [151, 152]. Health benefits of probiotics include increased production of SCFA, gut pathogens exclusion, enhanced epithelial barrier function, promotion of antimicrobial peptide function and secretory IgA, as well as stimulating anti-inflammatory cytokines and the induction of T_reg_ cells [79, 153]. The exact mechanisms behind these beneficial effects of probiotics are still unclear, however, it has been demonstrated that their efficacy varies depending on the microbial strain used and the administration dose [154].

Probiotics have been given attention for their wide range of clinical uses not only with respect to gastrointestinal and immune-mediated conditions, but also affective disorders such as depression and anxiety [25, 155–159]. By now, findings have shown that alterations in the gut microbiome composition through probiotic treatments can have a direct or indirect impact on mental health through multiple mechanisms along the gut-brain-axis [160–163]. As empirical findings have already outlined the significant role of altered immune functioning and microbiome dysbiosis in the pathophysiology of depression and anxiety [66, 89], probiotics characterized by their anti-inflammatory and immune-regulatory features may constitute a promising add-on treatment for comorbid mood disorders in IBD.

A meta-analysis by Ng et al. [164] showed that probiotic supplementation was associated with a significant mood improvement in individuals with mild-moderate depression, but not in healthy individuals. In contrast, an older meta-analysis [165] including 5 randomized clinical trials found significant effects of probiotics on mood in patients with depression as well as in healthy individuals. With respect to depression and anxiety disorders, clinical data suggest that *Bifidobacterium longum* can regulate host immunity by changing the composition of the gut microbiome and thereby alleviating symptoms related to these psychiatric disorders [31]. In healthy individuals, a treatment with *Lactobacillus helveticus R0052* and *B. longum R0175* resulted in lower levels of psychological distress and depression compared to matched controls without this treatment [26, 27]. In addition, *Lacticaseibacillus rhamnosus* and *Enterococcus faecium* have been found not only to possess anti-inflammatory activity but also to regulate tryptophan metabolism [166–168], which in turn plays a crucial role in the pathophysiology of depression.

Findings suggest an improvement of psychological symptoms after an administration of e.g. *Bifidobacterium longum* [15, 31] and *Lacticaseibacillus casei* [32]. These positive effects seem to be underpinned in the modulation of immune functioning via enhanced production of various immunomodulatory metabolites such as SCFAs, known to support immunological tolerance and maintain inflammatory equilibrium [169]. In addition, animal studies have demonstrated positive effects of probiotic supplementation, resulting in a reduced intestinal permeability, normalization of HPA hyper-reactivity and stress perception [170–172]. These results indicate a potential treatment effect of probiotics to prevent the development of depressive symptoms by normalization of HPA axis activity.

Another approach that has shown promising results in promoting a healthier gut microbiome is the use of prebiotics. These provide microbes with sufficient nutrients so that they can colonize, resulting in a higher microbial diversity in the gut. Inulin-type fructan prebiotics have been shown to increase the numbers of beneficial bacteria such as Bifidobacterium spp.*, F. prausnitzii* and Lactobacillus spp. [173, 174], while at the same time reducing pathobiont *E. coli* adherence to epithelial cells [175]. Prebiotic interventions can result in increased production of SCFAs [176, 177], having beneficial effects for host’s health as already described above. Compared to the numerous prebiotic studies done in animal research, only few interventional studies have been performed in patients with IBD. So far, findings have indicated that a prebiotic treatment has a positive effect on disease activity in patients with CD [178, 179]. Further, administration of prebiotics was linked to a reduced production of pro-inflammatory cytokines [179–181] and reduced faecal calprotectin [182]. However, studies have delivered partially inconsistent results, with some studies demonstrating an increase of Bifidobacterium spp. in IBD [178, 180, 181] and other indicating no effect on Bifidobacterium spp. or *F. prausnitzii* after prebiotics treatment [183, 184]. With respect to psychological symptoms, a meta-analysis based on 22 studies has demonstrated a positive effect of prebiotic treatments on reducing symptoms of anxiety [162]. These positive effects have been observed in individuals reporting mental health issues as well as in individuals with pre-diagnosed irritable bowel syndrome [185]. In addition, prebiotic supplementation (e.g. administration of galactooligosaccharide) was also shown to result in decreased waking cortisol levels and thus, to reduced stress perception, having significant positive effects on mental health in both healthy and clinical populations [28, 34, 186, 187]. With regard to fatigue, beneficial effects of synbiotic supplementation have been shown in post-infectious conditions in the wake of respiratory infections or Covid-19 [23, 29].

**Fig. 3:**
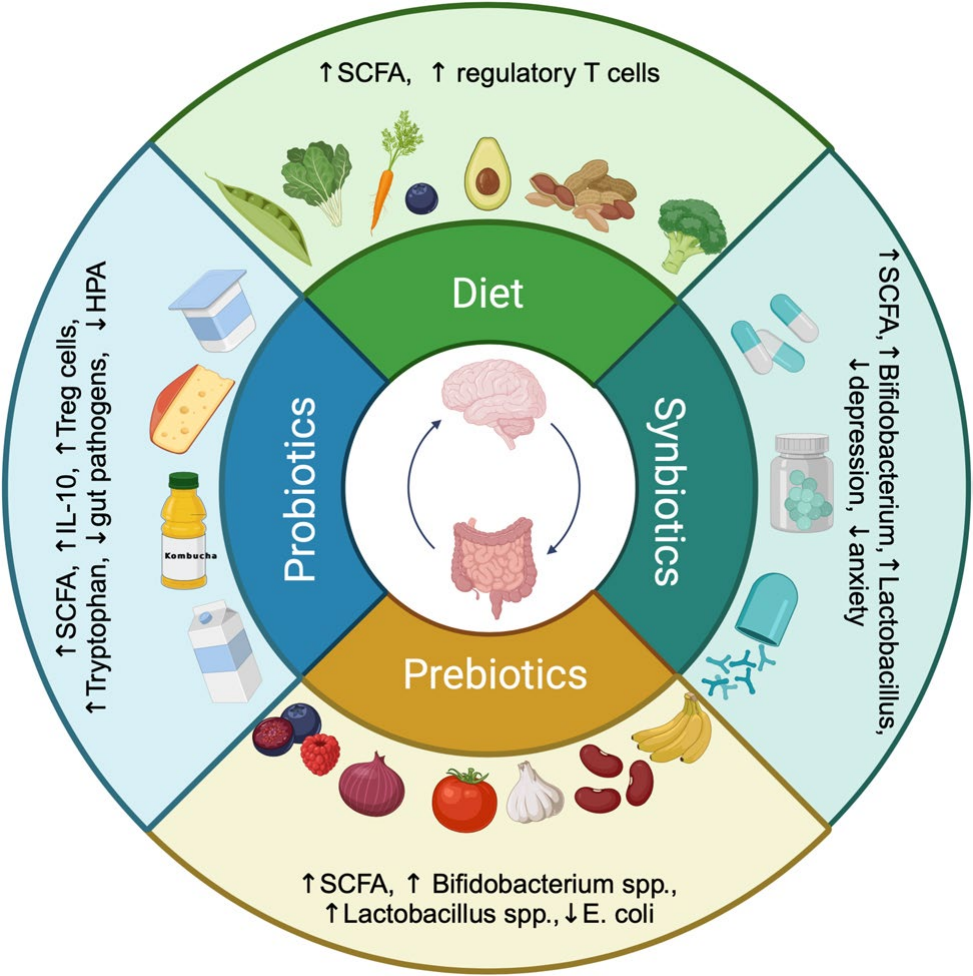
Immunonutrition, probiotic, prebiotic and synbiotic treatments as opportunity to modulate the gut microbiome in IBD by increasing the production of SCFA, downregulating the HPA axis and modulating tryptophan metabolism. Microbiome modulation can result in diminished inflammation, as well as reduced symptoms of depression, anxiety and fatigue in IBD. Figure created in https://BioRender.com

Microbiome modulation via pro- and prebiotics or the combination of both has been suggested as potential treatment opportunity for depression and anxiety symptoms as well as fatigue in IBD, but its therapeutic benefit has not been extensively explored in IBD populations. Based on a systematic literature research, a summary of available randomized clinical trials investigating effects of pro- and prebiotic supplementation on psychological wellbeing in clinical and healthy populations is available in Table 1. Given these encouraging results, the implementation of microbiome modulation by pre- and probiotics as a non-invasive treatment for psychological symptoms in IBD is a promising research area that warrants further study Fig. 3.

## Conclusion

Despite the significant increase in the number of studies exploring the link between IBD and neuropsychiatric symptoms as well as between psychiatric disorders and the gut microbiome in the last few years, there is still insufficient investigation into the role of the gut microbiome in the cause of impaired mental health in patients with IBD. There are several potential mechanisms through which alterations in the gut microbiome might affect IBD symptoms and especially symptoms of depression, anxiety and fatigue in this clinical population. Encouraging findings from other clinical populations suggest microbiome modulation as potential treatment option for comorbid neuropsychiatric symptoms in immune-mediated diseases. Future studies should specifically address patients with IBD and neuropsychiatric symptoms and include approaches that target the gut microbiome by dietary interventions or administration of pre-/pro- or synbiotics. Further, given the methodological limitations of available studies to date, high-quality RCTs are required to determine the effects of specific dose, treatment duration, and mechanisms of action to clarify how microbiome modulation can be used clinically to improve mental health and thus quality of life of patients with IBD.

## Supplementary Information

Below is the link to the electronic supplementary material.

Supplementary file1 (DOCX 40.4 KB)

## Data Availability

No new data were generated or analysed in this review.
