# Supplementary material for: Role of the gut microbiome in psychological symptoms associated with inflammatory bowel diseases
Source: Semin Immunopathol. 2025 Jan 27;47(1):12. doi: 10.1007/s00281-025-01036-x (PMC11772462; doi:10.1007/s00281-025-01036-x)
Supplement: Supplementary file 1 — Supplementary file1 (DOCX 40.4 KB) [file 281_2025_1036_MOESM1_ESM.docx]

**Supplementary material**

| **Animal studies** | | | | |
| --- | --- | --- | --- | --- |
| Abildgaard et al. (2017) | PRO | Sprague-Dawley rats | *Bifidobacterium bifidum, Bifidobacterium lactis, Lactobacillus acidophilus, Lactobacillus brevis, Lactobacillus casei, Lactobacillus lactis* | Reduction of depressive behaviors in the forced swim test |
| Arseneault-Bréard et al., 2012 | PRO | Rats with induced myocardial infarction | *Lactobacillus helveticus* and *Bifidobacterium longum* | Reduced depression symptoms related to post-myocardial infarction |
| Barrera-Bugueño et al., 2017 | PRO/PRE/  PRE + PRO | Sprague-Dawley rats | *Lactobacillus casei; inulin* | Anxiogenic-like effects after probiotics administration; reduction of anxiogenic effects after a subsequent prebiotic (synbiotic) administration |
| Bharwani et al., 2017 | PRO | C57BL/6 mice | *Lactobacillus rhamnosus* | Reduction of anxiety-like behaviors in social interactions |
| Cowan et al., 2016 | PRO | Rats exposed to maternal separation | *Lactobacillus rhamnosus, Lactobacillus helveticus* | No significant effect of probiotics on anxiety; beneficial effect on restoring emotion-related behaviors |
| Hao et al., 2019 |  | Sprague-Dawley rats exposed to chronic unpredictable mild stress | *Faecalibacterium prausnitzii* | Beneficial effects on depression- and anxiety-like behaviors |
| Jang et al., 2018 | PRO | Mice treated with immobilization stress | *Bifidobacterium adolescentis* | Reduced anxiety-like behavior |
| Liang et al., 2015 | PRO | SPF Sprague-Dawley rats | *Lactobacillus helveticus* | Reduced stress-induced anxiety and depression-like behavior |
| Liu et al., 2016 | PRO | Mice exposed to maternal separation | *Lactobacillus plantarum* | Reduced depression-like behavior but no significant effects on anxiety |
| Luo et al., 2014 | PRO | Rats with induced hyperammonemia | *Lactobacillus helveticus* | Reduced anxiety-like behavior |
| Messaoudi et al. (2011) | PRO | Rats and healthy volunteers | *Lactobacillus helveticus* and  *Bifidobacterium longum* | Reduced anxiety-like behavior in rats; decreased levels of psychological distress in volunteers, indicated by lower levels of depression, somatisation, and anger-hostility |
| Moya-Pérez et al., 2017 | PRO | C57B1/6J exposed to maternal separation | *Bifidobacterium pseudocatenulatum* | Lower anxiety levels after probiotic induction and attenuated stress response |
| Wang et al., 2015 | PRO | Rats with ampicillin-induced inflammation | *Lactobacillus fermentum* | Reduced anxiety-like behavior |

**Table S1.** Experimental animal studies on the effects of pre-/probiotics supplementation on depression- and anxiety-like behavior.

1. Abildgaard, A., et al., *Probiotic treatment reduces depressive-like behaviour in rats independently of diet.* Psychoneuroendocrinology, 2017. **79**: p. 40-48.

2. Arseneault-Bréard, J., et al., *Combination of Lactobacillus helveticus R0052 and Bifidobacterium longum R0175 reduces post-myocardial infarction depression symptoms and restores intestinal permeability in a rat model.* British Journal of Nutrition, 2012. **107**(12): p. 1793-1799.

3. Barrera-Bugueño, C., et al., *Anxiogenic effects of a Lactobacillus, inulin and the synbiotic on healthy juvenile rats.* Neuroscience, 2017. **359**: p. 18-29.

4. Bharwani, A., et al., *Oral treatment with Lactobacillus rhamnosus attenuates behavioural deficits and immune changes in chronic social stress.* BMC Med, 2017. **15**(1): p. 7.

5. Cowan, C.S., B.L. Callaghan, and R. Richardson, *The effects of a probiotic formulation (Lactobacillus rhamnosus and L. helveticus) on developmental trajectories of emotional learning in stressed infant rats.* Transl Psychiatry, 2016. **6**(5): p. e823.

6. Hao, Z., et al., *Faecalibacterium prausnitzii (ATCC 27766) has preventive and therapeutic effects on chronic unpredictable mild stress-induced depression-like and anxiety-like behavior in rats.* Psychoneuroendocrinology, 2019. **104**: p. 132-142.

7. Jang, H.M., et al., *Anxiolytic-like effect of Bifidobacterium adolescentis IM38 in mice with or without immobilisation stress.* Benef Microbes, 2018. **9**(1): p. 123-132.

8. Liang, S., et al., *Administration of Lactobacillus helveticus NS8 improves behavioral, cognitive, and biochemical aberrations caused by chronic restraint stress.* Neuroscience, 2015. **310**: p. 561-77.

9. Liu, Y.W., et al., *Psychotropic effects of Lactobacillus plantarum PS128 in early life-stressed and naïve adult mice.* Brain Res, 2016. **1631**: p. 1-12.

10. Luo, J., et al., *Ingestion of Lactobacillus strain reduces anxiety and improves cognitive function in the hyperammonemia rat.* Sci China Life Sci, 2014. **57**(3): p. 327-335.

11. Moya-Pérez, A., et al., *Bifidobacterium CECT 7765 modulates early stress-induced immune, neuroendocrine and behavioral alterations in mice.* Brain Behav Immun, 2017. **65**: p. 43-56.

12. Wang, T., et al., *Lactobacillus fermentum NS9 restores the antibiotic induced physiological and psychological abnormalities in rats.* Benef Microbes, 2015. **6**(5): p. 707-17.
